# Authors’ Reply: Safety of combination therapy of azilsartan medoxomil and amlodipine: a population-based cohort study

**DOI:** 10.4178/epih.e2025054

**Published:** 2025-09-20

**Authors:** Hyesung Lee, Bin Hong, Chris Tzu-Ting Su, Sungho Bea, Han Eol Jeong, Kyungyeon Jung, Michael Chun-Yuan Cheng, Zoe Chi-Jui Chang, Edward Chia-Cheng Lai, Jongyoung Lee

**Affiliations:** 1 Department of Medical Informatics, Kangwon National University College of Medicine, Chuncheon, Korea; 2 School of Pharmacy, Sungkyunkwan University, Suwon, Korea; 3 School of Pharmacy, Institute of Clinical Pharmacy and Pharmaceutical Sciences, College of Medicine, National Cheng Kung University, Tainan, Taiwan; 4 Division of Pharmacoepidemiology and Pharmacoeconomics, Department of Medicine, Brigham and Women’s Hospital and Harvard Medical School, Boston, MA, USA; 5 DSC Investment, Seoul, Korea; 6 Division of Cardiology, Kangbuk Samsung Hospital, Sungkyunkwan University School of Medicine, Seoul, Korea

Dear Editor,

We appreciate the thoughtful letter from Dr. Zhanyi Zhou regarding our recent study [1] on the safety of azilsartan medoxomil and amlodipine combination therapy in patients with hypertension. We thank the author for emphasizing the clinical importance of evaluating safety, specifically in patients with hypertension and concomitant heart failure.

While our study rigorously adjusted for multiple comorbidities, including chronic liver disease, chronic obstructive pulmonary disease, diabetes, and cerebrovascular and coronary artery disease, we acknowledge that heart failure was not included as a baseline covariate. This limitation was due to the lack of information on key clinical variables, such as New York Heart Association functional class and ejection fraction. However, given the new-user, active-comparator design and the robust adjustment for numerous relevant confounders, we expect that the prevalence and impact of heart failure would have been comparable between the treatment groups [2]. Furthermore, the relatively small sample size in the azilsartan medoxomil group and the anticipated low proportion of patients with heart failure limited our ability to perform a meaningful subgroup analysis by heart failure status in this study.

Considering the central role of angiotensin II receptor blockers in the management of both hypertension and guideline-directed heart failure, explicitly evaluating safety outcomes in patients with hypertension and concomitant heart failure would indeed provide valuable clinical guidance [3]. We fully support the recommendation that future studies incorporate such subgroup analyses to enhance the generalizability and clinical relevance of safety data across both general and high-risk populations.
